# Artificial intelligence in adolescents mental health disorder diagnosis, prognosis, and treatment

**DOI:** 10.3389/fpubh.2023.1110088

**Published:** 2023-03-31

**Authors:** J. Andrew, Madhuria Rudra, Jennifer Eunice, R. V. Belfin

**Affiliations:** ^1^Computer Science and Engineering, Manipal Institute of Technology, Manipal Academy of Higher Education, Manipal, Karnataka, India; ^2^Electronics and Communication Engineering, Manipal Institute of Technology, Manipal Academy of Higher Education, Manipal, Karnataka, India; ^3^Electronics and Communication Engineering, Karunya Institute of Technology and Sciences, Coimbatore, Tamil Nadu, India; ^4^BRIC, School of Medicine, University of North Carolina, Chapel Hill, NC, United States

**Keywords:** artificial intelligence, machine learning, deep learning, mental health, adolescent, neuroimaging

## 1. Introduction

Social, psychological, and emotional wellbeing are all considered to be components of one's mental health. It affects how someone thinks, feels, and responds to circumstances. When one has good mental health, it is easier to perform efficiently and reach their full potential (1). Preschool, adolescence, and adulthood are all included in the definition of mental health. Anxiety, social phobia, depression, panic disorder, substance dependence, and specific illnesses are factors that contribute to mental health problems that result in mental illness. The mental health status of adolescents in India is a topic of great concern and importance. Adolescents are children within the age group of 10–19. According to the National Mental Health Survey of India (2015–2016) (2), the prevalence of psychiatric disorders among adolescents of ages 13–17 is 7.3% and in the US it is 27.9% (3). This problem is further aggravated by stigma and lack of awareness surrounding mental health and a treatment gap of 95% in common mental disorders which is greater than the treatment gap for severe disorders (76%).

Early diagnosis of mental health issues is a crucial step for improving patient care and understanding of mental health diseases (4). However, the diagnosis of mental disorders is challenging because sometimes it is hard to distinguish between mental and physical health. The diagnosis of mental illnesses is reliance on a person's self-reporting to targeted interrogations used for the identification of particular patterns of public interactions and emotions. Unlike other chronic illnesses, this does not require laboratory testing or measures (5). While treating mental health problems, a challenge that is often faced is the lack of mechanistic models for psychiatric disorders. The complexity of the brain renders it difficult to have a clear model describing the development of a given mental illness (6).

The recent advancements in Artificial Intelligence (AI), Machine Learning (ML), and Deep Learning (DL) are widely applied in many domains and have made a successful dent in the healthcare sector too. The performance of AI, ML, and DL and based on the availability of data. Since mental health data availability is increasing every day AI techniques can be applied to better understand mental health (7). Recently in healthcare sectors, DL techniques provide superior performance in diagnosis, prognosis, and treatments. In medicine, AI is used in the form of machine learning models to understand medical data to gain insights in order to improve health outcomes and patient experience. Mental health professionals employ AI to fulfill various tasks such as providing insights into therapy sessions for better quality, refining diagnosis and monitoring patient's condition and altering treatment as deemed necessary (8).

## 2. Deep learning techniques

Deep Learning (DL) techniques are popular for their layered architecture that can learn the complex features of the data. DL techniques generally contains an input layer that takes input features, output layer that gives the learnt output and multiple hidden layers finds the relationship between the input and output classes. All layers perform the mathematical computations to find the relationship between various patterns of the input and output data. In this section, some of the popular DL techniques are introduced.

### 2.1. Neural networks

Neural networks are also called Artificial Neural Networks (ANN) that mimic human brain neurons. Each neuron is can perform a mathematical operation. The human brain has millions of neurons that are interconnected together. The main idea of ANN is to mimic the human brain like activity. Artificial neurons forward the weighted sum of the inputs through an activation function such as sigmoid, rectified linear units (ReLU) and so on for the non-linear transformation of the data. A simple ANN consists of a single hidden layer where DNN (Deep Neural Networks) consists of multiple hidden layers. The number of hidden layers increase the complexity of the network and it is helpful when the data is complex. The other variants of neural networks are feed forward neural networks, and back propagation neural networks.

### 2.2. Convolutional neural networks

Convolutional Neural Network (CNN) is a type of neural network, that comprises of convolutional operations and neural network layers (9). CNN is basically designed for image classification where it takes the image pixels as input and performs convolution and pooling operations to learn the low and high level features from the images and correspond them to the output-label. The basic architecture of a CNN contains a convolution layer, pooling layers, and fully connected (FC) layers. Pooling layers are used for edge detection and feature detection through max, min and average pooling operations. FC layer is a neural network where each neuron is connected to all the neurons in the next layers and forms a network. CNN models yield superior performance with image data hence, CNN can be used in the diagnosis of mental disorder through medical image analysis or through facial recognition. The variants of CNN models are Conv1D, Conv2D, and Conv3D.

### 2.3. Recurrent neural networks

Recurrent Neural Networks (RNN) are specifically designed for data that has temporal features. RNN maintains the temporal relationship between the features by feeding them to a recurrent neuron. RNN can extract features and long-term dependencies from sequential and time-series data (10). LSTM (Long Short Term Memory) and GRU (Gated Recurrent Units) are some of the well-known RNN models. LSTM can be used to capture the dynamic temporal features of brain signals for the diagnosis of mental disorders. GRU is less complex compared to LSTM so based on the size of the dataset the type of RNN model can be selected.

### 2.4. Generative networks

Generative models have gained attention for their ability to create synthetic data. As the success of the DL techniques are purely based on the availability of the data, generative networks help in creating synthetic data when there is less data availability, or to treat missing values and useful in data augmentation. Generative Adversarial Network (GAN), Variational Autoencoders (VAE) are some of the popular generative models (11). GAN is a combination of generator and discriminator networks where the model can recreate a look-a-like data from the learned features. VAE has encoder and decoders in the network, the encoder learns the latent spatial information from the input data and the decoder reconstructs the original data from the learnt representation.

## 3. Mental disorders in adolescents

Among adolescents, anxiety disorders and mood disorders are the most common. While anxiety and mood issues are two to three times more prevalent in female adolescents than in male adolescents, attention deficit disorder affects male and female adolescents differently. Adolescent patients of obstetricians and gynecologists are more likely to have one or more mental health issues than younger patients (12). The following are some of the common mental disorders.

### 3.1. Anxiety disorders

The most common anxiety disorders are General Anxiety Disorder (GAD) and panic disorder, which affect 3.6% of 10–14-year-olds and 4.6% of 15–19-year-olds, respectively. Excessive anxiety and disproportionate worry over a variety of things are symptoms of GAD.

### 3.2. Mood disorders

According to estimates, mood disorders such Major Depressive Disorder (MDD) and bipolar disorder impact 2.8% of teenagers aged 15–19 and 1.1% of adolescents aged 10–14. MDD is defined as a minimum 2-week period marked by either a loss of interest in previously enjoyed activities or a melancholy mood.

### 3.3. Attention deficit hyperactivity disorder

Attention Deficit Hyperactivity Disorder (ADHD) occurs among 3.1% of 10–14 year-olds and 2.4% of 15–19 year-olds. It is characterized by maladaptive and inconsistent with developmental level symptoms of inattention, hyperactivity, or impulsivity.

### 3.4. Obsessive compulsive disorder

An individual's obsessions or compulsions can lead to obsessive compulsive disorder (OCD). Obsessions are characterized as unwelcome intrusive ideas that recur frequently in a person's head. Compulsions, on the other hand, are compulsive mental patterns of thought or behavior that one feels compelled to engage in.

## 4. Diagnostic tools

Typically, mental diseases are identified based on a person's self-report on certain questionnaires made to look for certain emotional or social interaction patterns. The Diagnostic and Statistical Manual of Mental Disorders, Fifth Edition (DSM-5) is the diagnostic instrument that is most frequently used. A youngster or teenager may find it challenging to maintain concentration for more than 2–3 h during such conventional diagnostic interviews. Therefore, neuroimaging, which captures brain activity, serves as an alternative to such interviews. The diagnosis of various types of mental illnesses in adolescents is aided by understanding of brain activity.

### 4.1. MRI

Magnetic resonance imaging (MRI), a non-invasive imaging procedure, produces precise, three-dimensional anatomical images (13). Functional MRI (fMRI) and Structural MRI (sMRI) are the two types of neuroimages that are used for diagnosing mental disorders. Human brain activity changes are captured in fMRI data as Blood Oxygenation Level Dependent (BOLD) signals. The structured representation of the brain is shown in 3D in sMRI data together with its spatial configurations and voxel intensities. In state-of-the-art research, DL techniques are successful in analyzing neuroimage data.

### 4.2. EEG data

Electroencephalogram (EEG) signals are electrical signals that represent brain activity. EEG signals can be recorded using small low-cost sensors. The sensors record the electrical signals generated by the brain. EEG signals are instrumental in diagnosing various types of health disorders. It also plays a major role in diagnosing mental disorders.

### 4.3. PET

A functional imaging technique called a positron emission tomography (PET) scan employs radioactive materials called radiotracers to track and evaluate changes in physiological processes like blood flow, regional chemical composition, and absorption. PET helps in the development of diagnostic biomarkers for mental health diseases.

### 4.4. MEG

Magnetoencephalography (MEG) is similar to EEG that records brain activity through electrical signals. MEG is superior to EEG which has better spatial resolution with less artifacts. MEG can also be used for the diagnosis of psychiatric disorders.

### 4.5. Genetic data

Numerous common and uncommon genetic variations, such as single nucleotide polymorphisms (SNPs), have been linked to mental health issues using conventional statistical research in genetics and genomics, such as genome-wide association studies. Recent advances in next-generation sequencing technologies have generated a large amount of high-throughput genome or exome sequencing data, enabling researchers to examine patients with mental health illnesses by examining a variety of genetic changes across an individual's genome.

## 5. AI for mental disorder diagnosis

Artificial Intelligence has been found to be advantageous in the process of diagnosis of medical disorders. With the application of various techniques involving machine learning such as Boltzmann machine, support vector machine (SVM), K-Nearest Neighbor (kNN), diseases are detected and diagnosed (14, 15). Machine learning and computer vision, which are subsets of AI, are widely used due to their imaging, segmentation and predictability capabilities. Machine learning approaches are successful due to their focus on the performance of the models on new data which is also known as generalizability. Computer vision is applied in the process of diagnosis by detecting, segmenting and classifying images e.g., segmentation of radiological images and then further classifying them into diagnostic categories, detection of presence of metastases etc. Schizophrenia is serious mental disorder where a person is in hallucinations or delusion and interprets reality differently. To diagnose such disorder, Khan et al. (16) proposed a deep neural network which takes the genome sequencing data as the input and learns the feature representation of the data to diagnose the Schizophrenia disorder. The proposed model has attained an area under the curve (AUC) of 0.57.

AI is also widely applicable in diagnosis of mental health conditions (17). The diagnosis of a new patient is predicted using the training dataset of the diagnosis of the previous patients. Furthermore, artificial intelligence can also differentiate between diagnosis of diseases with similar symptoms but divergent methods of treatment. This is observed in the instance when bipolar or unipolar depression (18) is to be identified based on brain imaging features and types of dementia are to be differentiated based on structural MRI scans (19). Data-driven AI methods based on various factors such as demographic features, neurocognitive and biomarker profiles can aid in identifying novel disease subtypes (20). Moreover, AI methodologies can decipher patterns from data stemming from a long time span, which is critical for validating the accuracy of diagnoses based on the evolution of psychiatric conditions over a span of time. Sen et al., utilized a generative network called autoencoder that learns the spatial latent representation of the data. The model worked with fMRI and sMRI data for the diagnosis of ADHD. The model used multimodal features of the input to improve the classification accuracy up to 67%.

## 6. AI for mental disorder prognosis

The strong predictive power and generalization ability of AI is useful in the prognosis of mental disorders. AI techniques can utilize psychiatric patient's longitudinal data that is observed over a period of time for accurate prognoses. The longitudinal data can be neuroimaging data, genetic genomic data, and electronic health records (EHR). Koutsouleris et al. (21) collected MRI and genotyping data of psychiatric patients with high-risk syndromes and recent onset depression for the prognosis of psychosis mental disorder. For the prediction of psychosis disorder SVM technique is utilized which performs the classification of high-risk, recent-onset, and normal control from the learnt features from multimodal data.

Another type of brain data that can be used to perform the prognosis of mental disorder is EEG signals. In Ref. (22), Zhdanov et al., have used resting state EEG signals to predict the effectiveness of escitalopram treatment for the patients with depression. The authors used radial basis function (RBF) SVM to estimate the treatment effectiveness there by performing a prognosis study of the patients. The proposed model had achieved an accuracy of 82.4% for the classification among different severities of the disorders. Geng and Xu (23) and Nieuwenhuis et al. (24) analyzed the neuroimaging data using DL techniques such as autoencoder and CNN. They utilized fMRI and sMRI data to perform prognosis study of patients with depression and Schizophrenia disorders. Smucny et al. (25) studied the clinical improvement of psychosis patients from the fMRI data. A variety of ML and DL models are used to compare the performance of the techniques on the prognostic study is presented. The author has observed that multilayer perceptron or neural networks have performed well on the fMRI data for the accurate prognosis of psychosis patients with an accuracy of 70% and MSE (Mean Squared Error) of 9.47.

## 7. AI for mental disorder treatment

AI can be effectively used to measure the response to various treatments. Also, it can play a role in predicting the response of various drug combination which can help to develop precision medicines. Chang et al. (26) have measured the effectiveness of methylphenidate for treating ADHD from neuroimaging biomarkers. A multivariate pattern recognition approach is developed to measure the differences in the volumetric information in the sMRI. SVM is used as the classifier to perform the binary classification between the two classes of different volumetric measures. Li et al. (27) measures the gray matter volumetric (GMV) correlates in adolescents ADHD. Machine learning models are utilized to classify the normal and malignant samples of sMRI. Koutsouleris et al. (28) have used neuroanatomical pattern classification to predict the mental disorder transition. Psychosis (at-risk mental states) subjects sMRI data are collected and classified using SVM. The different classes for the classifiers are early at-risk, late at-risk, and healthy controls. The model achieved an accuracy of 82%in classification.

CNN-based DL models are efficient in classifying neuroimaging data. Acharya et al. (29) utilized EEG signals for automated depression screening. The authors have proposed a deep CNN model to classify the EEG signals of normal and depressive subjects. The model considered EEG signals of left and right hemisphere of the brain. The performance of the model was 93% and 95% respectively. Zou et al. (30) proposed a 3D CNN model to learn the low level and complex features form fMRI and sMRI neuroimaging data for ADHD treatments. The model was able to achieve an accuracy of 69.15% with limited training samples. Table 1 shows an overview of the various AI techniques in mental disorder diagnosis, prognosis, and treatment for neuroimaging data.

**Table 1:** AI techniques for mental disorder diagnosis, prognosis, and treatment.

**Reference**	**Application**	**AI technique**	**Mental disorder type**	**Dataset type**	**Performance**	**Strength/weakness**
Mikolas et al. (31)	Diagnosis	Support vector machine (SVM)	Schizophrenia	sMRI	ACC: 62.34% (*p* = 0.005)	Early detection of mental health disorders
Uyulan et al. (32)		CNN	Depression	EEG	ACC: 92.66% AUC: 0.9	Potential biomarker for confirming mood disorders
Drysdale et al. (33)		Clustering	Depression	fMRI	ACC: 89.2%	Helps to identify individuals who can benefit from neurostimulation therapies
Luo et al. (34)		Ensemble model	ADHD	Multimodal	ACC: 78.3% AUC: 0.89	Potential neurobiological markers for neurodevelopmental disorders
Zou et al. (35)		3D CNN	ADHD	fMRI	ACC: 65.67%	Potential neurobiological markers for neurodevelopmental disorders.
Khan et al. (16)		DNN	Schizophrenia	Genome sequencing data	AUC: 0.57	Identification of genes that causes mental disorder
Yan et al. (36)		LSTM	Schizophrenia	fMRI	ACC: 83.2%	Potential deep-chronnectome-learning with time courses
Koutsouleris et al. (21)	Prognosis	SVM	Depression	Multimodal	ACC: 85.9%	Prediction of psychosis transition
Geng and Xu (23)		Autoencoder and CNN	Depression	fMRI	ACC: 0.95	Considered 8 causality measures for depression prognosis
Sheynin et al. (37)		DNN	Post-traumatic stress disorder (PTSD)	fMRI	ACC: 81.3% AUC: 0.84	Single patient characterization is lacking
Nieuwenhuis et al. (24)		CNN	Schizophrenia	sMRI	ACC: 89% (*p* < 0.001)	Lack of large homogeneous data for multi-center prognosis
Smucny et al. (25)		Neural networks	Schizophrenia	fMRI	ACC: 70% MSE: 9.47	Potential biomarker for schizophrenia and psychosis treatment
Koutsouleris et al. (28)	Treatment	SVM	Psychosis	sMRI	ACC: 82%	Potential biomarker for prodromal phase of psychosis
Chang et al. (26)		SVM	Depression	fMRI and sMRI	ACC: 87.4% (*p* < 0.001)	Lack of multimodal data consideration that can improve the model efficiency
Acharya et al. (29)		CNN	Depression	EEG	ACC: 0.935	Different stages of depression is not explored
Zou et al. (30)		CNN	ADHD	fMRI and sMRI	ACC: 0.692	Potential biomarker for ADHD disorders
Pinaya et al. (38)		Autoencoder	Schizophrenia	sMRI	ACC: 0.639–0.707	Efficient in calculating neuroanatomical deviations in neuropsychiatric populations

## 8. Limitations of AI in mental health

The clinical validity and readiness for implementation of AI applications in clinical decision-making and patient care are both constrained. The performance of any AI application is affected by the quality and size of the data, an example of which is overfitting which is caused due to small and limited sample sizes. The generalizability of ML models is further restricted by testing them only using data from the same sample and not out-of-sample. The input features such as clinical data and demographics also restrict the predictive capability of such researches. Since no study is exhaustive enough to consider all factors, the clinical efficiency of the features used to derive the models must be considered. The validity of the outputs of the algorithms can be applicable only to a certain group of people or in specific situations (39).

Binary classifiers are more commonly used instead of regressors due to the ease of training them. However, using this approach neglects the severity of a condition. Hence, studies in future should take into consideration the severity of the condition. Another challenge that is faced by studies is modeling rare events or illnesses of highly imbalanced datasets. For such instances, classifiers predict the majority. However, this can be overcome by methods such as over-sampling and under-sampling. Over-sampling involves matching ratios of the minority and majority by duplicating minority samples meanwhile under-sampling involves reducing the number of majority samples. There are also ensemble learning methods which are combining several methods in order to reduce variance and improve predictions.

Moreover, mental health treatment relies on soft skills like rapport and relationship building with the patients and observing their emotions and behavior. The absence of crucial components like human compassion and empathy is also a hindrance in the treatment process. Further research is necessary on AI applications to prevent it from working in an unpredictable manner. Extensive risk assessment and administrative oversight is required before being put into medical use as it should be able to handle unusual situations.

Patient privacy is at risk due to the increased use of online servers. Online databases and the devices they are stored at are vulnerable to hacking and unauthorized monitoring. In India, the Ministry of Health and Family Welfare released a draft of Healthcare Security Act, which penalizes breach of data and promotes maintenance of electronic records. However, there is still a lack of guidelines regarding the assistance received by mental health professionals on delivering AI services. There are no laws defined to hold the software developer accountable for an technological glitches. Its other drawbacks include, most prominently the lack of human empathy and compassion, which are crucial components when treating patients who have suffered mental trauma or are experiencing a mental condition.

### 8.1. Future directions

AI struggles to self-reflect and account for the diversity amongst people, their thoughts, perspectives and morality. To deal with the human compassion and empathy limitations of AI, the concept of Artificial Wisdom has been introduced. Wisdom is associated with greater societal and individual wellbeing. The idea of creating AI that has human societal values and wise is something that is yet to be explored. It is unlikely that human wisdom will ever be fully programmed into machines, but partial instances are present in the form of robots being physical therapists and social workers along with providing cognitive assistance to the elderly (40). These developments are along the direction of creating machines who employ wise principles to make wise decisions. The advancements of AI wisdom can play a major role mental healthcare in the future.

## Author contributions

JA and MR conceived the idea. JA, MR, JE, and RVB devised the work, the main conceptual ideas, the proof outline, and worked on the manuscript. JA and MR worked on the technical details. All authors contributed to the article and approved the submitted version.
